# New Frontiers in the Pathobiology and Treatment of Cancer Regimen-Related Mucosal Injury

**DOI:** 10.3389/fphar.2017.00354

**Published:** 2017-06-08

**Authors:** Marika Cinausero, Giuseppe Aprile, Paola Ermacora, Debora Basile, Maria G. Vitale, Valentina Fanotto, Giuseppe Parisi, Lorenzo Calvetti, Stephen T. Sonis

**Affiliations:** ^1^Department of Oncology, University and General HospitalUdine, Italy; ^2^Department of Oncology, San Bortolo General HospitalVicenza, Italy; ^3^Divisions of Oral Medicine, Brigham and Women’s Hospital, BostonMA, United States; ^4^Dana-Farber Cancer InstituteBoston, MA, United States; ^5^Biomodels LLC, WatertownMA, United States

**Keywords:** gastrointestinal mucositis, oral mucositis, pathobiology, anticancer treatment, management

## Abstract

Mucositis is a common complication of chemotherapy, radiotherapy and targeted agents. It often affects compliance to anticancer therapies as it frequently causes schedule delays, interruptions or discontinuations of treatment. Moreover, the economic impact related to the management of mucositis is topical and several estimations of additional hospital costs due to this clinical condition have been recently reported. The ability to determine risk factors for mucositis, to early detect its onset, to assess correctly the degree of this toxicity and to plan its multidisciplinary management are all key elements to guarantee the quality of life of patients and to avoid useless dose reduction or interruption of treatment. The pathogenesis of mucositis is multifactorial and it is classily subdivided into oral and gastrointestinal mucositis according to its anatomic presentation. Treatment and patients’ related factors might help in predicting the frequency and the potential degree of symptoms onset. Here we discuss about clinical presentation and pathogenesis of mucositis in relation to different kinds of treatments. Moreover, we focus on therapeutic and prevention strategies, describing past and present management according to international guidelines and the most promising new data about agents potentially able to further improve the treatment of mucositis in the next future.

## Introduction

Mucositis is a common and clinically significant side effect of both anticancer chemotherapy (CT) and radiation therapy (RT) that can affect any portion of the gastrointestinal (GI) tract. Not only it is associated with an adverse symptom profile, but also it may limit patients’ ability to tolerate treatment if not adequately prevented and managed. Moreover, it may be associated with secondary local and systemic infection and poor health outcomes, and generates additional use of healthcare resources resulting in additional costs (Villa and Sonis, 2015).

Historically, mucositis has been described by its anatomical distribution: oral mucositis (OM) for involvement of the tissues of the upper aerodigestive tract, gastrointestinal mucositis (GIM) for lesions dominantly in the small intestine, and proctitis for injury of the rectal mucosa. The incidence and course of mucositis is site-dependent and related to the cancer treatment regimen.

OM has been the most studied, probably as a consequence of its frequency, ease of access and its course and symptom impact (Sonis et al., 2004). Nonetheless, all forms of mucositis (as well as other epithelially based toxicities) share common features in a complex scheme of pathogenesis. While the historical paradigm suggested that mucosal injury was solely the consequence of damaging effects of CT or RT on rapidly dividing normal cells of the GI tract, more current research has demonstrated that tissue damage occurs as a manifestation of a sequence of biological events that ultimately target epithelial stem cells. Experimental evidence has accumulated to validate mucositis’ pathogenesis as a multi-stage process (Sonis, 1998, 2004).

## Epidemiology

Like most other toxicities, the incidence of mucositis is likely to be under-reported by clinicians. The incidence of clinically significant mucositis has been reported to range from 15% among patients receiving low-risk treatments up to 60–100% among patients being treated with high-dose CT, radiotherapy and bone marrow transplantation. Nonetheless, this percentage is estimated to be about 40% in patients undergoing standard dose, cycled CT (Kwon, 2016). The incidence range of oral and non-oral mucositis at fixed doses of CT ranges from the single digits to well over 50% (i.e., TPF induction regimens for the treatment of HN cancer). Antimetabolites, anthracyclines, and taxanes are chemotherapeutic drugs frequently associated with the development of mucositis (Pico et al., 1998).

Chemotherapy-induced diarrhea, the key clinical sign of GIM, was reported to occur in 89% of patients treated with FOLFIRI and 50% of patients treated with FOLFOX for colorectal cancer (Keefe et al., 2014). Concomitant use of total body RT in hematopoietic stem cell transplant (HSCT) conditioning regimens markedly increased mucositis throughout the GI tract. RT-induced diarrhea in patients being treated for HN or lung cancers was noted in 29% of patients treated with radiation alone and 42% of patients treated with concomitant CT-RT.

Overall, almost a half million patients will suffer from mucositis this year in the U.S. with a likely similar number in Europe (Sonis et al., 2015).

Both OM and GIM can adversely impact on patients’ quality of life and may cause treatment delays, unplanned interruptions or even premature discontinuation of anticancer therapies, resulting in prolonged hospital stays, increased re-admission rates, more complications and economic burden. It has been reported an estimated incremental cost of hospitalization that may exceed 3,500 USD per cycle with mucositis (Elting et al., 2003) and an incremental cost of about 18,000 USD in HN cancer patients undergoing CT-RT (Nonzee et al., 2008).

## Pathobiology of Mucositis

**FIGURE 1:**
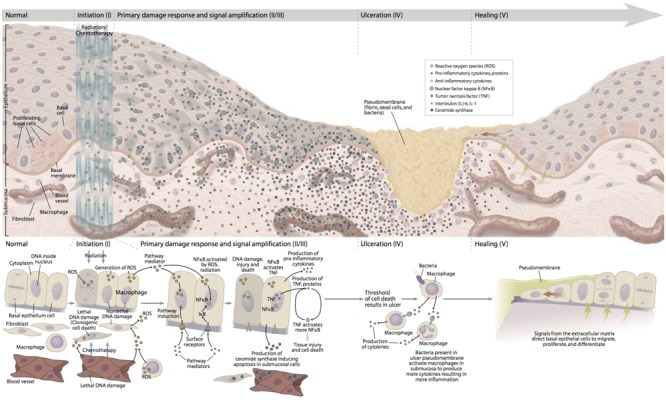
Pathobiology of mucositis. With permission of Sonis (2007). “Reprinted with permission from Frontline Medical Communications, Inc.”

The pathogenesis of mucositis is multifaceted and involves not only the epithelium, but also the cells and tissues within the submucosa (**Figure 1**). Signaling from damaged endothelium, fibroblasts and infiltrating leukocyte cells contributes to apoptosis, loss of renewal, atrophy and ulceration. Whereas these changes occur more slowly in stratified mucosa, they are abrupt in the single layers of the small intestine (Chaveli-López, 2014; Villa and Sonis, 2015).

A five-phase sequence has been used to describe the biological phases of mucositis: initiation, up-regulation and activation leading to generation of messengers, signal amplification, ulceration with inflammation, and healing. For the most part, this order is independent of the insult (RT and CT) or the target tissue involved. Importantly, the elements driving each phase represent potential interventional targets (Sonis et al., 2004).

The initiation of mucositis is triggered by oxidative stress and the generation of reactive oxygen species (ROS), direct DNA and non-DNA damage, and activation of the innate immune response. These events follow the release of endogenous damage-associated molecular pattern molecules from injured cells of the basal epithelial layers, submucosa, and endothelium. Based on the trajectory of gene activation and pathway analysis, it is clear that the initiating biological cascade happens within seconds of the stimulating insult.

Following initiation, ROS and the innate immune response further damage cell membranes, stimulate macrophages and activate several transcription factors of which nuclear factor NF-κB plays a prominent role. Once activated, NF-κB-mediated gene expression results in a surge of many pro-inflammatory cytokines such as tumor necrosis factor-α (TNF-α), interleukin (IL)-6 and IL-1β and cyclooxygenase-2 (COX-2). The up-regulation of other genes causes the expression of adhesion molecules and angiogenesis (Sonis, 2002; Rm et al., 2007).

More in depth, TNF-α up-regulation may activate caspase pathways and generate a feedback on NF-κB to amplify its response and initiate mitogen-activated protein kinase (MAPK) pathway, leading to activation of c-Jun N-terminal kinase (JNK) signaling; fibronectin breakdown leads to macrophage activation. NF-κB independent pathways such as ceramide pathway may also play a role, resulting in apoptosis of submucosal and basal epithelial cells leading to mucosal ulceration (ulcerative phase) and atrophic changes. Recent studies confirmed the involvement of deregulated expression of metalloproteinases (MMPs) in the pathobiology of mucositis (Al-Dasooqi et al., 2010).

The first three phases rapidly lead to apoptosis of epithelial stem cells. In the case of stratified epithelium (i.e., the upper aerodigestive tract and rectal mucosa), loss of renewal leads to atrophy and then ulceration. From the clinical point of view, the overlying mucosa appears initially normal despite the biological havoc taking placing beneath it. In the case of bolus CT, the time between initial basal cell injury and clinical notable mucosal changes (erythema and thinning) takes about 4 days with ulceration occurring shortly thereafter. In contrast, the consequences of cellular damage in the intestinal villi are almost immediate with clinical evidence of enteritis becoming apparent within 24–48 h of CT.

Bacterial colonization of non-intestinal lesions lags slightly behind ulcer development. However, at that time, a large increase in the bacterial load is seen. In the case of patients receiving CT, this occurs at the time that the patient is least capable of dealing with potential infection as it roughly inversely parallels the course of the leukopenia. Ulcer colonization also results in the release of bacterial cell wall products and cytokine production. Healing generally occurs spontaneously and is characterized by epithelial proliferation, migration, and differentiation stimulated by the extracellular matrix (Blijlevens and Sonis, 2007; Al-Ansari et al., 2015). After the healing phase, the oral mucosa returns normal, although the patient have an increased risk of future episodes of mucositis due to residual angiogenesis.

### The Role of Microbiota

An active role for the oral and intestinal microbiome in the course of mucositis has not been conclusively established. Antimicrobial strategies aimed at mitigating mucositis have been unsuccessful; moreover, the kinetics of the bacterial load seem to follow, rather than lead ulceration development. Nonetheless, it would be naïve to believe that bacteria are simply inert once lesions are colonized. Certainly, we know that cell wall products that easily penetrate disrupted mucosa have the ability to stimulate macrophages to produce pro-inflammatory cytokines (Stringer and Logan, 2015).

In the GI tract, cancer treatments may affect the composition of luminal microbiota. Generally, they cause a decrease in *Lactobacillus* and other protective bacterial species and an increase in specific pathogenic species (Stringer et al., 2013).

Probiotic bacteria may activate cytoprotective pathways in epithelial cells, counteract ROS, displace pathogenic bacteria and interact with tight junctions to enhance mucosal integrity (Ciorba, 2012). Determining a role for bacteria in intestinal mucositis is further complicated by the observation that GIM is most often manifest in the small intestine, an area of the GI tract in which bacteria are markedly less dense than in the colon (Ciorba et al., 2015). Nonetheless, bacterial transmigration across disrupted epithelium provides an opportunity for bacteremia or systemic infection.

A role for oral viruses, particularly herpes simplex, in the patho-etiology of mucositis, especially in myeloablated patients has been considered for years. Likewise, the potential role for *Candida albicans* in the mucositis development has also been considered (Chen et al., 2011). Based on clinical presentation, cellular data around pathogenesis, and the consistent observed failure of anti-viral or anti-fungal prophylaxis to mitigate mucositis, attribution of mucositis to an infectious etiology is highly unlikely.

### Chemotherapy-Induced Mucositis

Although different CT drugs may target different parts of the cell cycle or metabolism, their effect on intestinal morphology is consistent and characterized by decreased crypt length, blunting and fusion of villi, enterocytes hyperplasia and increased apoptosis. The small intestine is most often affected. Commonality of aspects of mucositis pathogenesis is also noted, although the lack of uniform study endpoints hinders some comparisons across different classes and specific agents. A role for pro-inflammatory cytokines has been suggested by a number of studies of both 5-FU, methotrexate and irinotecan (Logan et al., 2008, pp. 1139–1145) in which TNF, IL-1β, and IL-6 levels were all elevated prior to tissue changes (Logan et al., 2008). Likewise, proteins associated with apoptosis (i.e., Bcl-2) regulation are impacted by a range of cytotoxic agents (Ribeiro et al., 2016).

Irinotecan, a topoisomerase I inhibitor, has been broadly studied relative to mucositis pathogenesis. Results of an extensive series of animal studies confirm similarities of cancer regimen-related GI injury pathobiology with that suggested for OM including roles for tight junction disruption and matrix metalloproteinase-mediated connective tissue damage (Wardill et al., 2014; Chen et al., 2016). Likewise, irinotecan-induced mucositis is associated with the activation of caspases, p53 and downregulation of the PI3K/Akt pathway (Mayo et al., 2017), activation of the MAPK and PKC pathways.

The specific anatomy of the small and large intestine contribute to the establishment of mucositis as a ‘downstream event.’ For example, reduction in goblet cells number and mucin hypersecretion likely contribute to the development of diarrhea.

Some evidences suggest that GIM may manifest in two different ways during irinotecan treatment. Early-onset diarrhea is due to the activation of parasympathetic system leading to cholinergic syndrome by the inhibition of acetylcholinesterase or the release of large quantities of acetylcholine. On the other hand, late-onset diarrhea appears to be multifactorial with both cytokines and direct toxic inflammatory-mediated effects on the mucosa as well as motility alteration (Ribeiro et al., 2016).

Likewise, the development of mucosal injury in platinum-based CT is associated to the mucin reduction.

### Radiotherapy-Induced Mucositis

In the case of radiation, damage signaling at the cellular and tissue level happens within seconds of exposure. While the biological sequence is similar to that described above, the fractionated schedule of radiation dosing insures continuing and overlapping damage signals and tissue change. In the case of the upper GI tract and rectal mucosa, symptoms associated with atrophic changes (burning and modest pain) begin as soon as the end of the first week of dosing when patients have typically received 10 Gy of radiation. Ulceration is noted between the second and third week of treatment and becomes contiguous and extremely painful (so as to limit function) at cumulative radiation doses of 30–40 Gy. Lesions persist for up to 6 weeks following the completion of RT (Villa and Sonis, 2015).

Some authors have examined the role of p16 on mucositis and dysphagia incidence rate and duration in HN cancer patients undergoing RT plus cetuximab or RT alone. They have demonstrated that the addition of cetuximab is not related to higher incidence or duration of grade 3 or 4 mucositis compared to RT alone. Finally, they have also seen that patients with p16 negative seem to develop more frequently grade 3 or 4 mucositis (Bonner et al., 2016).

Interestingly, Bossi et al. (2016) found that baseline salivary cytokines levels in HN cancer patients undergoing CT-RT were not associated with severity of OM. However, the salivary concentration of IL-6, TNF-α, IL-10, and IL-1β tend to increase because of anticancer treatment especially during the third week, and it seems to be associated with mucositis severity. Particularly, higher IL-6 and IL-1β levels predicted the development of severe oral toxicity. On the other hand, osteopontin is very high at baseline and decreases after CT-RT (Bossi et al., 2016).

Meirovitz et al. (2010) previously showed that high levels of IL-6 and low levels of IL-8 were associated with percutaneous endoscopic gastrostomy (PEG) placement.

### Targeted Therapies-Induced Stomatitis

Among the targeted therapies currently used in oncologic practice, the mTOR-inhibitors produce the most consistent mucosal toxicity. While other sites in the GI tract may also be affected, the severity and impact of mTOR-inhibitor-associated stomatitis (mIAS) is most profound.

Because of its role as a central modulator of extracellular and intracellular signaling of mediators and growth factors associated with negative tumor behaviors, the mTOR pathway has become an attractive target for a class of targeted anti-tumor agents. mTOR-inhibitors, such as everolimus, are currently being used in the management of a number of solid tumors including breast, neuroendocrine of the GI tract and renal cell cancers. Of patients receiving these agents, about 40% develop severe ulcerative stomatitis, termed mIAS which is phenotypically similar to aphthous stomatitis (Peterson et al., 2015). Clinically, mIAS differs from conventional mucositis. mIAS lesions are seen on the movable oral mucosa appearing as relatively shallow, disproportionately painful, ulcers surrounded by an erythematous halo. The central portion of the ulcers is grayish reflecting an area of necrosis. Pseudomembranes are atypical and histologically lesions present as non-specific ulcers. The course of the mIAS is unpredictable, but ulcers can be manifest as soon as 5 days after the start of treatment (Sonis et al., 2010; Elting et al., 2013; Bossi et al., 2015). It appears that the pathogenesis of mIAS is associated with direct epithelial injury followed by a second inflammatory phase. In a recent study using an organotypic model of oral mucosa, histologic changes of mIAS were noted in the absence of any microorganisms. Increases in apoptosis and a reduction in cell proliferation based on immunohistochemical outcomes were seen as were changes in keratinocyte-derived pro-inflammatory cytokines. *In vivo* it is likely that the latter act to attract and facilitate the infusion of inflammatory cells (Sonis et al., 2016).

## Risk Factors for Mucositis

It is clear to anyone treating cancer patients that the risk of any toxicity, including mucositis, is not consistent. While some patients sail through treatment, others suffer immensely, despite having similar tumors and equivalent treatment. Given the imperatives of the Precision Medicine Initiative and the Cancer Moon Shot, prospective identification of patients at risk for mucositis is an important ongoing research objective. Understanding the mechanisms and incidence rates of GIM is essential to set an effective treatment avoiding treatment discontinuation that could negatively influence patients’ outcome. In general, risk factors may be associated with the treatment regimen and/or the patient (Villa and Sonis, 2015).

### Treatment’s Related Variables

Treatment’s related factors are linked to the type of anticancer treatment (CT, RT, targeted therapy, etc.), the agent used, the dose and schedule of the anticancer drug, agent or radiation (Sonis, 2010; Villa and Sonis, 2015, 2016).

#### Chemotherapy

The rates of onset and severity of mucosal injury depend on the type of CT used (Shi et al., 2016).

Chemotherapeutic agents vary in their mucotoxicity. For example, the antimetabolites, i.e., 5-FU (Schwab et al., 2008; Abdel-Rahman et al., 2016) and methotrexate, irinotecan (Stein et al., 2010; Mayo et al., 2017), alkylating agents like cyclophosphamide, and cisplatin (Villa and Sonis, 2015), and anthracyclines and taxanes (Kwon, 2016) all tend to be more consistently associated with mucosal toxicities than bleomycin, hydroxyurea, or etoposide. Moreover, bolus infusion tends to be more toxic.

Recently, a meta-analysis by Abdel-Rahman et al. (2016) has shown that the fluoropyrimidine S-1 induced lower risk of mucositis compared to 5-FU. Instead, patients treated with capecitabine had the same toxicity profile of S-1. The combination of fluoropyrimidines and irinotecan is associated with increased risk of GIM (Abdel-Rahman et al., 2016), especially when capecitabine is used. In the BICC study (Skof et al., 2009) patients who received XELIRI reported higher rates of severe diarrhea (∼50%) compared to patients exposed to FOLFIRI.

Moreover, some preclinical studies have found that ileal mucosa is more sensitive to the cisplatin than the remaining GI tract (Yamamoto et al., 2013). Despite this, patients with lung and GI cancer receiving platinum salts and 5-FU have a low risk of platinum-associated severe mucositis (Al-Ansari et al., 2015). Notably, GI toxicity induced by oxaliplatin and carboplatin tend to have a lower grade compared to that of cisplatin (Hartmann and Lipp, 2003).

Patients treated with taxanes experience mucositis in approximately 29–63% of cases. Interestingly, taxanes-associated mucosal damages usually are mild or moderate. Grades 3 and 4 occur only in a few percentage of patients. Furthermore, mucositis occurs more often in patients who receive docetaxel than paclitaxel.

In general, if a patient develops mucositis in the first cycle of treatment, the probability of the condition recurring in a subsequent cycle is high in the absence of dose de-escalation. Mucosal toxicities also arise due to an physiologically driven “overdosing.” Patients with hepatic and renal impairment may have a reduced clearance of antineoplastic drugs, which could potentially lead to a greater exposure to these agents.

#### Radiotherapy

Not surprisingly, patients being radiated for treatment of HN cancers are at high risk for OM (Trotti et al., 2003; Sonis, 2013). In fact, about two-thirds will develop severe forms of the condition. The incidence jumps to close to 100% for cancers located in the mouth or oropharynx.

The addition of concomitant CT, most typically cisplatin, is associated with an increased mucositis risk (Trotti et al., 2003). Sanguineti et al. (2012) showed that HN cancer patients receiving CT-RT had a 4.1-fold and a 5.1-fold increased risk of mucositis development when using IMRT and conventional RT fractions, respectively. It appears that both the incidence and duration of OM is increased with the addition of cetuximab to a standard regimen of RT when compared to CT-RT (*p* = 0.014) (De Sanctis et al., 2016).

Since radiation induces both direct and indirect injury, the observation that patients being treated for HN cancer also manifest damage to lower portions of the GI tract is not unexpected. The consequences of such lesions are impressive. Noteworthy, RT is often associated with the development of esophagitis. High-dose RT and concurrent CT results in significantly increased risk of severe esophagitis. Some patients may require a feeding tube and/or treatment interruptions. Furthermore, damage at this level may lead to superinfection and dysphagia or odynophagia, lower dietary intake, cachexia and consequently to worse prognosis (Adebahr et al., 2016). Radiation on pelvic or abdominal site leads to enteritis, which prevalence ranges from 0.5 to 50% (Abayomi et al., 2009; Theis et al., 2010; Webb et al., 2013). Small bowel-related complications are proportional to the volume of small intestine in the radiation field. Usually, this side effect was delayed, graded 1 or 2, with low rate of hospitalization. Obviously, toxicity was increased by the CT-RT combination therapy (Hernández-Moreno et al., 2015).

Finally, regimens using accelerated dosing schedules in which the daily cumulative dose exceeds 2 Gy are associated with an increased incidence and severity of mucositis.

#### Targeted Agents

As noted above, some forms of targeted therapy are associated with increased risk of mucosal injury. Since most of these agents, especially the biologicals such as cetuximab are given in conjunction with radiation, their specific mucotoxicity is difficult to assess. The combination of EGFR-I with RT or CT may further increase the toxicity. Notably, in the CRYSTAL trial, colorectal cancer patients randomized to receive FOLFIRI plus cetuximab showed higher frequency of grades 3 and 4 GIM than patients receiving FOLFIRI alone (Van Cutsem et al., 2009, 2011). The PRIME trial, randomizing patients to FOLFOX and panitumumab or FOLFOX alone, showed similar results (Douillard et al., 2014). Furthermore, FIRE-3 (Heinemann et al., 2014) and CALG-B (Venook et al., 2014) trials demonstrated that CT plus cetuximab induced more GI toxicities than CT plus bevacizumab (Aprile et al., 2015).

mTOR-inhibitors produce the most consistent stomatotoxicity of targeted agents and their incidence approaches or exceeds that observed with conventional cytotoxic agents (Elting et al., 2013; Rugo et al., 2014; Sonis et al., 2017). The related frequency and gravity depends on drugs doses and treatment duration, but mIAS is a common cause of dose-de-escalation or termination of treatment. Nonetheless, mIAS usually resolves spontaneously without treatment discontinuation (Boers-Doets et al., 2012). A meta-analysis (Abdel-Rahman and Fouad, 2015) evaluating the risk of oral stomatitis and enteritis in patients treated with everolimus, temsirolimus, and ridaforolimus showed an increased risk of toxicities compared to the control group. Median time of dose interruption was 7 days.

In the meta-analysis by Shameem et al. (2015), toxicities incidence and grade depended on cancer types independent of dose (*p* = 0.004). Particularly, renal cell carcinoma (RCC) were associated with fewer rate of mucositis (RR 1) than astrocytoma (RR 5.29), gastric cancer or breast cancer, regardless the combination of mTOR-inhibitors with other drugs. Furthermore, everolimus was associated with the highest risk of stomatitis (RR 4.5).

Mucosal damage caused by TKIs is associated with hypersensitivity and dysgeusia. OM occurs in 26% of sunitinib-treated patients and in 36% of patients receiving sorafenib (Lee et al., 2009). A meta-analysis on metastatic RCC showed that 81% of patients treated with sunitinib and 90% of those treated with sorafenib experienced AEs after 4 week of treatment. Dose reduction was required in 26% and in 18%, respectively (Boers-Doets et al., 2012).

In the CORRECT trial, regorafenib induced GI toxicity of any grade, among which diarrhea (34% vs. 8% in placebo arm) and OM (27% vs. 4%) were frequently reported (Grothey et al., 2013).

### Patient-Related Risk Factors

While a range of descriptive parameters have been indicated as predictors of mucositis risk including poor oral health, low body mass index, younger or older age, and female sex, none have been consistent and accurate. (Sonis et al., 2004; Chansky et al., 2005; Schwab et al., 2008; Sonis, 2010; Krishna et al., 2011; Chaveli-López and Bagán-Sebastián, 2016; Vasconcelos et al., 2016; Villa and Sonis, 2016). However, it now appears that identification of genomic drivers of pharmacokinetic and radio/pharmacodynamic factors which impact mucositis risk is possible through assessment of germline mutations, associated with those pathways affecting mucositis development or drug metabolism

The first genomic tests for toxicities were associated with the identification of mutations that impacted enzymes associated with drug metabolism.

For example, patients with deletion polymorphism of the thymidylate synthase (TYMS) gene (Cho et al., 2007) or dihydropyrimidine dehydrogenase (DPD) deficiency (Meulendijks et al., 2015) tend to have increased toxicity from 5-FU. However, the percentage of patients having even partial mutations of these genes is relatively small (<5% of the at risk population). Consequently, the impact of genomics on toxicity risk had to be more broadly based and associated with genes effecting those pathways involved in pathogenesis. This hypothesis has been confirmed for a number of regimen-related toxicities induced by both RT and CT. However, additional studies are mandatory to produce a working clinically applicable tool that can be routinely applied. The recent application of machine learning algorithms to this issue has accelerated the process.

## Clinical Presentation

Gastrointestinal mucositis can affect any site of the alimentary tract and it may present with a large spectrum of clinical manifestations according to the involved area (Al-Dasooqi et al., 2013).

The first clinical manifestation of OM is erythema of one or more sites of the movable mucosa (i.e., buccal or labial mucosa, ventral tongue, floor of the mouth or soft palate). Lesions typically progress to form painful ulcerations often covered by a pseudomembrane and accompanied by odynophagia, dysphagia, malnutrition, and weight loss (Peterson et al., 2012; Chaveli-López and Bagán-Sebastián, 2016). Disruption of the intact mucosa may be associated with microbial colonization that may remain localized or become disseminated, especially in patients with severe neutropenia (Chaveli-López, 2014; Chaveli-López and Bagán-Sebastián, 2016). OM is usually self-limiting and its course depends on the anticancer treatment. Among patients receiving CT, first signs appear shortly after administration and usually peak at about days 7–14 to completely recover within the following week (Al-Ansari et al., 2015; Villa and Sonis, 2015). On the other hand, RT-induced mucositis usually develops during the second or third week of treatment and often persist until 2–4 weeks after the last dose (Villa and Sonis, 2015).

Little data exist to accurately characterize the course of esophageal or gastric mucositis. Consequently, symptoms such as pain, dysphagia, dyspepsia, nausea, and vomiting are often attributed to gastroesophageal reflux or candidosis, leading to underestimate mucositis in this tract (Squier and Kremer, 2001; Aprile et al., 2015).

The onset of CT-associated intestinal mucositis (enteritis) tends to be acute (usually within 24–48 h after treatment) and may present with diarrhea, constipation, abdominal pain, nausea, vomiting, and anorexia. In some cases malnutrition, dehydration, infections, and sepsis may also occur (Al-Dasooqi et al., 2013; Ribeiro et al., 2016). Typhlitis, otherwise known as neutropenic enterocolitis, is a mucositis of ileo-cecal region with high mortality risk, typically affecting patients with neutropenic fever. Its clinical manifestation ranges from abdominal pain, bloating and diarrhea to acute abdomen (Davila, 2006). This severe form of enteritis may complicate treatments for hematologic tumors but it is observed also in patients undergoing cytotoxic drugs for solid malignancies (Sachak et al., 2015).

Proctitis usually occurs in patients undergoing chemoradiation for rectal, prostate or other pelvic cancers; symptoms include painful tenesmus with mucus discharge and rectal bleeding. Onset may be acute and/or not develop until several weeks after starting treatment. While these conditions are usually transient and resolve within a few weeks following the completion of RT, chronicity is not rare.

Moreover, both OM and GIM may cause systemic clinical manifestation such as anorexia, malabsorption, weight loss, anemia, fatigue, and sepsis (Al-Dasooqi et al., 2013).

In this landscape, targeted therapies-induced mucositis, such as mIAS, deserves a special mention and represents an emerging issue with different characteristics (Peterson et al., 2015; Kwon, 2016). According to ESMO guidelines (Peterson et al., 2015), the term stomatitis is more appropriate and should be used to indicate the mucosal inflammation related to these novel drugs.

## Assessment Scales

Assessment scales provide the basis of objective comparisons of regimen-related toxicities or efficacy of toxicity treatment intervention. Currently, there is not a single instrument which is used universally. Rather, a range of scoring instruments are used with each depending on somewhat different subjective and/or objective criteria to define the severity of GIM (Villa and Sonis, 2016). One of the most commonly used is the National Cancer Institute-Common Terminology Criteria for Adverse Events (NCI-CTCAE, most recent version 4.03), which grades mucositis severity 0–5, based primarily upon symptom severity, functional alteration and intervention requirements. NCI-CTC criteria for mucositis vary by anatomic site. The changing nature of NCI-CTC benchmarks, which has been a feature of each new iteration of the scale, has hindered longitudinal regimen-related toxicity comparisons (United States Department of Health and Human Services, 2010; Peterson et al., 2015). The World Health Organization (WHO) scale is widely used for grading OM and incorporates both objective and functional (ability to eat) assessments (Peterson et al., 2015; Villa and Sonis, 2015). Independently, the Radiation Therapy Oncology Group (RTOG) has developed Cooperative Group Common Toxicity Criteria which are, in some ways, a hybrid of those described by the NCI-CTC and WHO scores (Sonis, 2011).

Over the years, other scales designed primarily for use in clinical trials have been developed, such as the Oral Mucositis Assessment Scale (OMAS) (Sonis et al., 1999). However, measures developed for CT or RT-induced mucositis may not apply to patients treated with targeted agents. Thus, *ad hoc* scales have been designed for this population, such as the mIAS scale to assess mIAS (Boers-Doets and Lalla, 2013). Moreover, the integration with patient-reported outcome (PRO) becomes critical to improving the accuracy of clinical evaluation (Sonis, 2010; Bossi et al., 2015). Indeed, clinicians may underestimate the real burden of mucositis. Furthermore, the inter-observer variability can lead to discrepant scoring. Examples of PRO instruments are represented by the Oral Mucositis Daily Questionnaire (OMDQ) (Stiff et al., 2006b), the abovementioned OMAS and the Patient-Reported Oral Mucositis Symptom (PROMS) scale (Kushner et al., 2008).

It would be worthy to have a single standardized scale that incorporated clinicians and patients’ measures to describe GIM severity and to compare different prevention modalities and treatment regimens.

## Management of Gastrointestinal Mucositis: Current and Investigational Approaches

Although the quality of evidence derived from clinical studies is somewhat limited (Worthington et al., 2011), MASCC and ESMO have developed guidelines which offer potential strategies for managing mucositis (Lalla et al., 2014). It should be noted that the guidelines themselves are not definitive and represent the synthesis of a consensus of opinions of their authors. The guidelines should be viewed as fluid and will likely undergo changes as higher levels of evidence which support or refute treatment develop.

Notably, given the relatively recent development of new drugs, only expert opinions on the management of targeted therapies-induced mucositis are available (Peterson et al., 2015).

Prevention and treatment strategies for OM and GIM are listed in **Tables 1, 2**, respectively.

**Table 1:** Prevention and treatment strategies for oral mucositis.

Intervention	Aim	Clinical setting	Authors’ comment	Guidelines(grade of evidence)
Oral care protocols	Prevention	All cancer patients	General agreement on the value of oral care protocols	MASCC/ESMO (III) NCCN
Oral cryotherapy	Prevention	Bolus 5-FU chemotherapy	Safe, low cost, with some positive results	MASCC/ESMO (II) NCCN
		High-dose melphalan +/- TB-RT for HSCT	As above	MASCC/ESMO (III) NCCN
Palifermin	Prevention	High-dose CT and TB-RT for HSCT	Only approved agent for OM mitigation in a narrow patient population	MASCC/ESMO (II) NCCN ASCO
Low-laser therapy	Prevention	High-dose CT +/- TB-RT for HSCT	Data suggesting possible benefit	MASCC/ESMO (II)
		HN cancer patients receiving RT alone	Data suggests possible benefit, but potential tumor impact unresolved	MASCC/ESMO (III)
Benzydamine mouthwash	Prevention	HN cancer patients receiving moderate dose RT alone	Anti-inflammatory rinse with some data supporting its use in patients receiving radiation only	MASCC/ESMO (I)
0.2% morphine mouthwash	Pain treatment	HN cancer patients receiving CT-RT	Data suggests effective adjunct for topical pain control	MASCC/ESMO (III)
Doxepin mouthwash	Pain treatment	All cancer patients	Data suggests effective adjunct for topical pain control	MASCC/ESMO (IV)

*CT, chemotherapy; RT, radiotherapy; TB-RT, total-body radiotherapy, HN, head and neck; HSCT, hematopoietic stem cell transplantation; MASCC, Multinational Association of Supportive Care in Cancer; ESMO, European Society for Medical Oncology.*

**Table 2:** Prevention and treatment strategies for gastrointestinal mucositis.

Intervention	Aim	Clinical setting	Guidelines (grade of evidence)
Intravenous amifostine	Prevention of RT-induced proctitis	Patients receiving RT	MASCC/ESMO (II)
	Prevention of CT-RT-induced esophagitis	NSCLC patients	MASCC/ESMO (II) ASCO with reserve
Octreotide	Treatment of diarrhea	Standard or high-dose CT for HSCT	MASCC/ESMO (II)
Sucralfate enemas	Treatment of chronic RT-induced proctitis	Patients receiving RT with rectal bleeding	MASCC/ESMO (III)
Oral sulfasalazine	Prevention of RT-induced enteropathy	Patients receiving RT to the pelvis	MASCC/ESMO (II)
*Lactobacillus* probiotics	Prevention of diarrhea	Patients receiving CT +/- RT to the pelvis	MASCC/ESMO (III)
Hyperbaric oxygen	Treatment of RT-induced proctitis	Patients receiving RT for solid tumors	MASCC/ESMO (III)

*CT, chemotherapy; RT, radiotherapy; NSCLC, Non-small cell lung cancer; HSCT, hematopoietic stem cell transplantation; ASA, acetylsalicylic acid; MASCC, Multinational Association of Supportive Care in Cancer; ESMO, European Society for Medical Oncology.*

### Basic Oral Hygiene

Oral health at the start of and during cancer therapy appears to impact the course of OM. Consequently, oral care protocols which include pre-treatment comprehensive oral examination and elimination of sources of mucosal irritation and infection are crucial to prevent and reduce oral injury across all cancer treatment strategies (Peterson et al., 2015). Oral hygiene helps to reduce the bacterial load and, consequently, the infection risk (Rubenstein et al., 2004; Campos et al., 2014). It includes general hygiene standards, dental care, normal saline and baking soda mouthwashes, dietary and behavioral measures (Peterson et al., 2015; Mallick et al., 2016).

### Antioxidant Agents

Reactive oxygen species play a significant role in the pathogenesis of OM. Consequently, reducing their production or scavenging them from tissue is a potential interventional strategy. Antioxidant drugs may have a role in reducing mucositis through the suppression of ROS or the increasing of endogenous production of antioxidative enzymes (Ozben, 2015; Kwon, 2016).

#### Amifostine

It is a pro-drug of phosphorylated aminothiol and presents a cytoprotective action on salivary gland, decreasing Il-6 and TNF-α, protecting normal endothelium, connective tissue and gland tissue. The mechanism of action of this agent may be related to the recruitment of ROS scavengers, the protection of DNA and the induction of cellular hypoxia (Koukourakis and Maltezos, 2006). The use of this agent may have utility in preventing RT-induced proctitis, esophagitis, and OM (Lalla et al., 2014). With respect to OM, amifostine’s favorable effect on salivary gland function could also be beneficial in depressing OM course. However, intravenous administration and its unfavorable toxicity profile have limited amifostine’s utilization in routine clinical practice (Yuan and Sonis, 2014).

#### Glutamine

It is an amino-acid involving in glutathione synthesis. It acts across exhibiting antioxidant properties, particularly by accelerating mucosal remodeling (Tsujimoto et al., 2015). Results of studies assessing the efficacy of topical or systemic formulations of glutamine, a precursor of nucleotide synthesis, on the development and course of mucositis have been inconsistent.

#### Oral Zinc Supplement

This drug acts as an antioxidant through several functions, including epithelial proliferation, extracellular matrix synthesis and wound healing in damage tissue. Although the evidence supporting its use are relatively sparse (Arbabi-kalati et al., 2012; Van Sebille et al., 2015) and a mechanism of action is not completely clear, systemic zinc could be beneficial in the prevention of OM in oral cancer patients undergoing CT or CT-RT (Lalla et al., 2014).

#### Vitamin E

The efficacy of vitamin E as a mucositis intervention has been explored in animals and humans using different formulations. It is a α-tocopherol, that can limit tissue damage caused by therelease of ROS. The results of these studies has been inconsistent (Uçüncü et al., 2006; El-Housseiny et al., 2007; Ghoreishi et al., 2007; Azizi et al., 2015).

#### *N*-Acetyl-Cysteine (NAC)

This compound contains thiol groups. It is involved in antioxidant process by reducing the production of ROS, myeloperoxidase activity, as well as xanthine dehydrogenase and xanthine oxidase activity. Moreover, it participates in inflammation response, by activating of NF-kB. Moslehi et al. (2014) evaluated the efficacy of this glutathione precursor in a double-blind, randomized study in leukemic patients, showing a significantly lower OM rate in patients receiving NAC than patients receiving placebo. A rinse formulation of NAC was also shown to be effective in mitigating radiation-induced OM. In addition to its antioxidant properties, NAC’s mechanism also includes modulation of a variety of pathways known to important in mucositis pathogenesis including NF-κB.

#### Superoxide Dismutase Mimetics

Superoxide dismutase has been recognized as a potential interventional target. A phase 2 trial (NCT02508389) testing a superoxide dismutase mimetic is currently ongoing (ClinicalTrials. gov, 2017a).

### Inflammation and Cytokines Production-Inhibitors

#### Benzydamine

Benzydamine HCl is a non-steroidal anti-inflammatory agent in an oral rinse formulation. This anti-inflammatory effect is possible by inhibiting the production and the effect of pro-inflammatory cytokines, such as TNF-α. In addition, it has been shown that it has anesthetic, analgesic, and antimicrobial properties (Rubenstein et al., 2004). While it has demonstrated modest efficacy in patients with HN cancer being treated with RT in the absence of concomitant CT, it has been ineffective in attenuating OM in patients receiving standard combined regimens of cisplatin and radiation (Epstein et al., 2001; Kazemian et al., 2009; Sheibani et al., 2015).

#### Pentoxifylline

Pentoxifylline’s rationale as a mucositis intervention is based on its anti-TNF activity. It plays an important role in modulating inflammation, by inhibiting pro-inflammatory cytokines such as IL-1-β, TNF-α, and NF-kB. There is no evidence to support its use in clinical practice, although NCT02397486 trial is ongoing to evaluate the impact of pentoxifylline and vitamin E on mucositis in HN cancer patients receiving RT (ClinicalTrials. gov, 2016g).

#### Salicylates

A role for salicylates in the management of GIM is questionable. While sulfasalazine has been suggested to efficacious in attenuating RT-induced enteropathy in patients receiving pelvic RT, curiously acetylsalicylic acid, mesalazine or olsalazine are ineffective in preventing RT-induced diarrhea.

#### Interleukin Inhibitors

While pro-inflammatory cytokines appear to be a desirable target for mucositis prevention and treatment, clinical data assessing their use are sparse. A phase 2 trial (NCT01403064) failed to demonstrated efficacy of anti-IL-6 monoclonal antibody as an OM intervention (ClinicalTrials. gov, 2016f).

#### Other Biological Modifiers in Development

Given its complex pathogenesis, a number of mechanistically targeted agents are in various phases of development. Smad7, a TGFβ and NF-κB inhibitor has demonstrated interesting outcomes in animal models. Likewise, Antrum Mucosal Protein (AMP), which targets cell junctions and blocks endothelial and epithelial apoptosis effectively mitigated OM in an orthotopic mouse model (Chen et al., 2016). Favorable results of a Phase 2 study of an innate immune inhibitor (dusquetide) were recently reported (Kudrimoti et al., 2016). A proprietary topical formulation of clonidine successfully reduced the duration of OM in patients receiving concomitant CT-RT for HN cancer (Onxeo press release). Trefoil factor 1 released by genetically modified *Lactococcus lactis* bacteria was effective in decreasing the duration of OM in patients receiving induction CT as part of treatment regimen for HN cancer (Limaye et al., 2013). A phase 2 trial evaluating the defensin mimetic brilacidin is ongoing (ClinicalTrials. gov, 2017b).

### Cytoprotective Agents

#### Prostaglandin Analogs

Prostaglandin analogs have a cytoprotective action on mucosal tissue. More in depth, it stimulates the production of bicarbonate, mucous, blood flow with subsequently endothelial and epithelial cellular protection. Despite this background, it has not proved to be effective as a mucositis intervention (Lalla et al., 2012, 2014).

#### Sucralfate

Sucralfate is a basic albumin salt. It binds to proteins exposed by ulceration, providing a protective coat against the action of pepsin and gastric acid. Moreover, it stimulates the production of local prostaglandins, angiogenesis, and fibroblast proliferation. On the other hand, it inhibits the release of cytokines and it has antimicrobial activity. Therefore, it has thus been suggested to be potentially of value in palliating mucosal injury, particularly by generating granulation tissue and wound-healing process (Ala et al., 2016). However, clinical trial results using the compound have been conflicting (Lalla et al., 2014).

### Growth Factors

#### Palifermin

It is the recombinant human keratinocyte growth factor-1 (KGF-1) which belongs to fibroblast growth factors’ (FGF’s) family. It stimulates the proliferation and the differentiation of epithelials cells, but it has more stability than the analogous native protein, due to its particular structure (Rubin et al., 1989). In preclinical setting palifermin has showed defensive role in several epithelial tissues (Farrell et al., 1998).

This drug has also has pleotropic, antiapoptotic, antioxidant and anti-pro-inflammatory activity (Villa and Sonis, 2015). Intravenous infusion of KGF-1 successfully impacted the course of severity of OM in patients receiving aggressive stomatotoxic conditioning regimens prior to HSCT (Spielberger et al., 2004; Stiff et al., 2006a) and was subsequently approved by Food and Drug Administration (FDA) and European Medical Agency (EMA) for use restricted to this patient population. The efficacy of palifermin in other patient populations has not been sufficiently studied and its use in patients bearing tumors which themselves have KGF receptors has limited its more broad application.

#### Other Growth Factors

There is no consistent or compelling evidence to support the use of granulocyte-macrophage colony-stimulating factor (GM-CSF), granulocyte colony-stimulating factors (G-CSF, e.g., filgrastim) or FGF (Yuan and Sonis, 2014; Chaveli-López and Bagán-Sebastián, 2016; Mallick et al., 2016) as an OM mitigator. (Lalla et al., 2014).

### Antiapoptotic Agents

Apoptosis has been demonstrated to be critical for the development of OM (Kwon, 2016) so it is not unexpected that therapeutic antiapoptotic strategies have been considered. The finding that chemokine ligand 9 (CXCL9) exacerbated intestinal injury in a 5-FU animal model suggests that it might represent a viable therapeutic target (Han et al., 2011). Similarly, specific caspase-3 inhibition was protective in an animal model of RT-induced OM.

### Physical Strategies

#### Oral Cryotherapy

Several controlled trials provide evidence for the benefit of cryotherapy (ice chips) in modulating OM (Cascinu et al., 1994; Baydar et al., 2005; Sorensen et al., 2008; Riley et al., 2016). A recent Cochrane review concluded that oral cryotherapy probably reduces the severity of OM (RR 0.61, 95% CI 0.52–0.72) and the incidence of severe OM (RR 0.4, 95% CI 0.27–0.61) in patients undergoing FU-based treatment (Riley et al., 2015). It was hypothesized that cryotherapy’s benefit was derived from local vasoconstriction, leading to reduced exposure of the mucosa to FU (Chaveli-López and Bagán-Sebastián, 2016). A randomized-controlled, open-label, phase 1–2 NCT02326675 trial is ongoing to evaluate cryotherapy in the prevention of CT-induced mucositis in stem cell transplant (ClinicalTrials. gov, 2016b).

#### Laser Therapy (Photobiomodulation)

Several trials suggest that mucosal treatment with a low level helium-neon laser (LLLT) reduces the severity of mucositis and promotes healing in patients undergoing conditioning therapy for HSCT (Barasch et al., 1995; Cowen et al., 1997; Schubert et al., 2007; Ferreira et al., 2016). Similar trials have been performed in patients receiving RT alone for HN cancer (Lalla et al., 2014; Peterson et al., 2015). A significant amount of data exists documenting the robust biological activities of LLLT. As has been recently pointed out, many of the biological pathways activated by LLLT have been associated with poor tumor outcomes and/or resistance to treatment. Until there is definitive data establishing that LLLT is inert relative to tumor response and behavior the use of such therapy in areas of tumor is to be approached with caution (Sonis et al., 2016; Zecha et al., 2016).

### Pain Management

Pain management plays a crucial role in improving patient’s quality of life. To date, patient-controlled analgesia with morphine is recommended only in the treatment of OM-related pain in hematologic patients (Lalla et al., 2014). Transdermal fentanyl, morphine mouthwashes, and doxepin rinse are other possible options in various clinical settings (Lalla et al., 2014; Van Sebille et al., 2015). Tapentadol, gabapentin, and pregabalin are under investigation.

To date, magic or miracle mouthwash are also available; this term applies to a variety of rinses typically based on institution-specific formulations and folklore. They include various compounds of topical anesthetic (e.g., lidocaine), a muco-adherent vehicle and other agents such as antimicotics, steroids or antibiotics. Their efficacy is unproven (Chaveli-López and Bagán-Sebastián, 2016).

Moreover, a number of topical coating agents are currently available including GelClair^®^, Episil^®^, and MuGard^®^. Of these, the only MuGard^®^ has been evaluated in a prospective, randomized, placebo-controlled, blinded, multi-institutional trial and has shown palliative benefit (Allison et al., 2014). Nonetheless, there are reports of symptomatic benefits for the other agents (Yuan and Sonis, 2014; Villa and Sonis, 2016). Caphosol, a remineralizing solution, has been tested in multiple, randomized, blinded trials of which the results do not generally support its efficacy for an OM indication (Rao et al., 2014; Svanberg et al., 2015; Wong et al., 2016; Treister et al., 2017).

A recent exploratory study investigated the role of methylene blue, a type A inhibitor of monoamine oxidase acting on microglial cells that seem to be involved in neuroinflammation and pain control (Roldan et al., 2017).

### Other Management Approaches

#### Probiotics and Antimicrobial Agents

*Lactobacillus* species-containing probiotics may be of value in preventing diarrhea in patients undergoing CT and/or RT for pelvic tumors. Even if the mechanism remain unclear, in preclinical models probiotics seem to improve the crypts of small intestinal, preserving architecture and preventing some alterations of the goblet cell, such as the decrease of acidic mucin, after CT (Prisciandaro et al., 2011). NCT01707641 (ClinicalTrials. gov, 2016c) is an ongoing trial evaluating the preventive effect of *Lactobacillus* on RT-CT-induced OM in HN cancer patients, while NCT02819960 trial is investigating the role of probiotics in preventing irinotecan-induced diarrhea (Mego et al., 2015).

Antibiotic strategies using conventional or investigational agents have not proven to be efficacious in favorably impacting mucositis. Conflicting data exist about the use of chlorhexidine rinse (Dodd et al., 2000; Campos et al., 2014).

#### Dexamethasone Mouthwash

The preliminary results of the multicenter phase II SWISH trial suggest a benefit in managing mIAS (Rugo et al., 2016). If true, such an approach most likely targets the secondary inflammatory phase of these lesions, but a properly performed, randomize, placebo-controlled trial is currently lacking.

#### Glucagon-Like Peptide-2 (GLP-2) analogs

Several studies have suggested a potential role of these agents in treating irinotecan-induced mucositis and diarrhea. GLP-2 analogs have been demonstrated to limit and improve this toxicity in animal models.

### Natural Remedies

A number of organic agents are under investigation to determine potential preventive or therapeutic effect. Vitamin A, ascorbic acid (ClinicalTrials. gov, 2016d), manuka honey, aloe vera, chamomile, curcumin (ClinicalTrials. gov, 2016e), and other plant extracts (ClinicalTrials. gov, 2016a) are just some examples of an emerging approach (Yuan and Sonis, 2014; Van Sebille et al., 2015).

## Conclusion

Gastrointestinal mucositis remains a significant, common unmet clinical need in cancer patients. Although frequently reported, the real rate and impact of this worrisome toxicity may be underestimated, and it consistently contributes to burden in terms of negative impact on quality of life, outcome and healthcare costs. The baseline risk-assessment is crucial to identify patients more likely to develop severe GIM in order to provide the best possible preventive and therapeutic approaches, with the aim of preserving optimal treatment intensity and maximize patients’ safety.

To date, most of the literature reports refer to OM, while the management of GIM remains a major challenge. In recent years, the increasing knowledge on the mucositis pathobiology has provided opportunities for the development of new approaches based upon the underlying molecular pathways. Although an increasing number of possible treatments have emerged, no standard measures have been established. A future, biologically based strategy may consist in combining interventions acting on the different phases of mucositis’ pathogenesis.

More research efforts are needed to better understand the underpinning biological processes in order to develop new effective treatments. Investigations should be performed to further characterize the role of the oral environment, including studies on the potential contribution of the oral/periodontal microbiome in the pathobiology of mucositis associated with targeted agents. Similarly, studies on changes in salivary output and proteome induced by anticancer therapies may contribute to a scientific base for OM risk prediction, early diagnosis and interventions (Al-Ansari et al., 2015).

Despite its longstanding recognition, frequency, clinical impact and cost, the treatment options for mucositis are disappointingly sparse. Only one agent, palifermin, has been approved for mitigation of OM in the U.S. – and only for a very limited segment of the at-risk population. GI mucositis suffers a similar fate. Its management is reliant on symptom control.

Next year will mark two decades since the recognition that the biological basis for mucositis’ pathogenesis is far more complex than simply being ascribed solely to non-specific clonogenic cell death of epithelial stem cells. The presentation of that concept and data from the subsequent studies that have followed, provided a plethora of information which have had tremendous potential translational value in identifying druggable targets for the enablement of new drugs and biologicals. Consequently, as discussed above, we are seeing a broad range of mechanistically based compounds in all phases of pre-clinical and clinical development. Preventing or limiting CT- or RT-induced normal tissue injury, while not interfering with a desired anti-tumor effect is not easy. The development of animal models to both study the pathobiology of mucositis and to serve as pre-clinical development platforms has been critical. For the most part, animal models for mucositis have been rodent (mouse, rat, and hamster) based. While no model is perfect, the predictive value of these ones relative to assessing a compounds behavior in humans has been unquestionably valuable. For example, the efficacy outcomes of a number of compounds that were observed in hamster models has been replicated in human studies. Likewise, although accumulating data highlight the differences between normal and tumor cells behavior, assessing that medications for supportive care do nothing to hinder the effectiveness of cancer therapy or induce negative tumor behaviors is critical. Animal models have been conducted for this purpose prior to the start of clinical trials.

However, animal models are characterized by several limitations and some successes which may be present in pre-clinical setting are not always evident in the clinical one. First of all, few animal studies focus on GIM, with most of the evidence deriving from trials on OM (Bowen et al., 2005; Viet et al., 2014). Moreover, in some animal models mucosal ulceration requires mechanical injury (Sonis et al., 1990), while in human patients the development of mucositis is independent of mechanical irritation. Another issue of pre-clinical models is represented by doses and scheduling; indeed, the susceptibility to a particular chemotherapic or radiotherapic regimen may be different between species. In addition, some animal models are characterized by the need of higher doses of CT than humans to induce the same grade of mucositis, due to the different keratinization of the epithelium. Moreover, such models sometimes require a route of drug administration not translatable to human patients. A further issue derives from the use of fully humanized monoclonal antibodies, which may not be active in animal setting (Bowen et al., 2011). Finally, few pre-clinical trials exist in order to investigate the molecular pathways of mucosal pain and most of the evidence is derived from animal models of pain related to oral cavity tumors or temporomandibular disorders (Viet et al., 2014).

Continued development of models and robust analyses of how animal results compare with those in humans will provide the information needed to help optimize the pre-clinical pathway for the development of new therapies.

All of this is taking place in an environment which increasingly recognizes that patients differ in their individual risk for mucositis (and other toxicities) and in how they might respond to one treatment or another. As a result, the literature reflects studies which now embed concepts of precision medicine in clinical trial design for mucositis interventions.

Given the impact of the above on the trajectory and enthusiasm for developing effective preventive and treatment options for mucositis, it is hard not to be optimistic that the current pipeline will result in effective therapies for mucositis in the relatively near future.

## Author Contributions

Manuscript writing: MC, GA, PE, DB, MV, and SS. Final approval of manuscript: MC, GA, PE, DB, MV, VF, GP, LC, and SS.

## Conflict of Interest Statement

The authors declare that the research was conducted in the absence of any commercial or financial relationships that could be construed as a potential conflict of interest.
